# Analysis of macular microvasculature changes in human immunodeficiency virus infection using swept-source OCT angiography

**DOI:** 10.1186/s12886-026-04822-9

**Published:** 2026-04-18

**Authors:** Meryem Doukh, Mohamed Ghorbel, Maha Abid, Amir Bedhiafi, Amel Omezzine Letaief

**Affiliations:** 1 Department of Ophthalmology, Zaghouan Regional Hospital, Environnement Avenue, Zaghouan, Tunisia; 2 Department of Ophthalmology, Farhat Hached Hospital, Rue Ibn Jazzar, Sousse, 4031 Tunisia; 3 Department of Infectious Diseases, Farhat Hached Hospital, Rue Ibn Jazzar, Sousse, 4031 Tunisia; 4 Department of Medical Intensive Care, Fattouma Bourguiba Hospital, Rue 1er juin, Monastir, 5000 Tunisia

**Keywords:** Optical coherence tomography angiography, Retinal blood flow, Retinal imaging analysis, Human immunodeficiency virus, HIV retinopathy

## Abstract

**Background:**

To evaluate macular optical coherence tomography angiography (OCTA) parameters and microvascular alterations in Human Immunodeficiency Virus (HIV)-positive patients, with and without clinically detectable HIV-related non-infectious retinal vasculopathy.

**Methods:**

This comparative study included HIV-positive patients and age- and sex-matched healthy controls. All participants underwent multimodal retinal imaging, including color fundus photography, fluorescein angiography, and macular OCTA. Quantitative OCTA parameters—parafoveal vessel density (VD) of the superficial (SCP-VD) and deep capillary plexuses (DCP-VD), as well as foveal avascular zone (FAZ) area, perimeter, and circularity—were analyzed. Qualitative microvascular changes were also assessed.

**Results:**

A total of 102 HIV-positive patients and 100 healthy controls were included. All six eyes with clinically detectable HIV retinopathy demonstrated perifoveal capillary abnormalities on OCTA. Among HIV-positive eyes without funduscopic or angiographic evidence of retinopathy, 72 eyes (75%) showed microvascular changes on en face OCTA. Mean SCP-VD and DCP-VD were significantly reduced in the HIV group compared to controls (*p* < 0.001 and *p* = 0.004, respectively). FAZ was significantly larger and less circular in HIV-positive eyes (*p* < 0.001). Multivariate analysis revealed that longer duration of HIV infection correlated significantly with OCTA vascular changes (*p* = 0.033).

**Conclusion:**

OCTA enables early detection of subclinical retinal microvascular alterations in HIV-positive patients, even in the absence of clinically apparent retinopathy.

**Supplementary Information:**

The online version contains supplementary material available at 10.1186/s12886-026-04822-9.

## Introduction

The human immunodeficiency virus (HIV) pandemic has affected approximately 80 million people worldwide since 1981 [1]. Sight-threatening ocular manifestations can be observed in 30 to 75% of HIV-positive patients during the disease course [2]. The advent of highly active antiretroviral therapy (HAART) resulted in a lower ocular manifestations, now affecting approximately 15 to 45% of HIV-infected individuals [2].

Previous reports have described various ophthalmic clinical presentations, but HIV-associated retinopathy was the most identified ocular manifestation that occurs more commonly in patients with lower CD4 + lymphocyte count and higher HIV plasma viral load. It is characterized by focal ischemia affecting small vessels, associated to clinically detectable cotton wool spots, intraretinal hemorrhages, and microaneurysms. HIV-related microvasculopathy has been associated with neurocognitive disorders, accelerated organs aging and has been identified as an independent prognostic factor for mortality [3–5].

Optical coherence tomography angiography (OCTA) is a relatively new non-invasive imaging technique that has enabled precise analysis of retinal and choroidal circulation. It detects endoluminal flow and generates high-resolution images that provide quantitative metrics including vessel density (VD) layer-by-layer [6, 7]. OCTA can also reveal subtle microvascular alterations that that may be missed with fundus examination or conventional fluorescein angiography (FA) [8].

In this study, we aimed to assess the macular OCTA parameters and microvasculature changes in HIV-positive patients, with and without clinically detectable HIV-related non-infectious retinal vasculopathy.

## Materials and methods

This prospective, cross-sectional, case-control study carried out among HIV patients recruited in both infectious diseases and ophthalmology departments of Farhat Hached University Hospital in Sousse (Tunisia), during 6 months period from January 2023 to June 2023. The present study was conducted following the principles stated in the 1964 Helsinki Declaration and received approval from the research and ethics committee of Farhat Hached University Hospital (ID number of the approval: CER 31-2022). All patients included in the study provided written informed consent prior to their participation.

### Participant recruitment

Patients diagnosed with HIV infection were initially included. The diagnosis was confirmed using the Western blot test. Patients with a medical history of diabetes mellitus, age-related macular degeneration, hypertensive retinopathy, or any congenital or acquired ocular pathology that could potentially influence retinal vasculature were excluded. Presence of any opportunistic ocular infection was also considered as an exclusion criterion. Subjects with a refractive error between − 3D and + 3 D or having poor quality imaging resulting from any ophthalmic pathology that may interfere with retina visualization such as corneal opacity, cataract or vitreous opacity were excluded from the study.

Patients with or without detectable HIV microvasculopathy (assessed clinically and through FA) were included in the study. HIV microvasculopathy was diagnosed based on the presence of detectable cotton-wool spots, intraretinal hemorrhages, microaneurysms, and telangiectatic vessels on fundus imaging and/or FA.

A group of age and gender-matched healthy participants was recruited as controls to compare their OCTA findings with those from the HIV group.

For patients with HIV infection, information regarding time from the diagnosis, current medications, duration of HAART, HIV RNA plasma level, and CD4 + T cell count was noted. Virologic failure in HIV is characterized by a confirmed viral load greater than 50 RNA copies/ml after at least 6 months of effective ART.

### Ophthalmologic assessment

All patients and controls underwent complete ocular examination including best-corrected visual acuity (BCVA) converted to logMAR, spherical equivalent (ES), intraocular pressure (IOP), slit-lamp biomicroscopy of the anterior segment and dilated fundus examination.

### Retinal imaging acquisition

Swept source OCT (SS-OCT) device (DRI OCT Triton Series; Topcon, Tokyo, Japan) was used for macular imaging. SS-OCT uses a central wavelength of 1,050 nm, axial resolution of 8 μm, lateral resolution of 20 μm, and a scan speed of 100,000 A-scans/s.

All OCTA measurements were made after pupil dilation. En face OCTA images of 3 × 3 mm and 4.5 × 4.5 mm centered on the fovea were acquired in all patients. Rescan was conducted if the image quality was dissatisfied with apparent flaws. Scans with image quality index « IQI » > 50 were further evaluated. In addition, areas of the fundus with HIV microvasculopathy, such as cotton-wool spots, were scanned. Macular structural B-scan OCT was also performed in all patients. Color fundus photography and fluorescein angiography images were performed using a retinal camera: Kowa VX-10i Fluorescein Angiography (Kowa Company, Ltd., Nagoya, Japan).

### Retinal imaging analysis

Two independent trained researchers (M.D and M.G) have separately analyzed the results. Images were analyzed using Triton ImageNET 6 (software version 1.22). Two-dimensional OCT-A images were generated using the following predefined axial slabs: superficial (from 2.6 μm below the internal limiting membrane (ILM) to 15.6 μm below the inner plexiform layer plus the inner nuclear layer (IPL/INL)), deep (from 15.6 μm below IPL/INL to 70.2 μm below IPL/INL), outer retina (from 70.2 μm below the IPL/INL to Bruch’s membrane (BM)), and choriocapillaris (from BM to 10.4 μm below BM).

All en face OCTA slabs at the level of SCP, DCP and choriocapillaris were automatically segmented. In the 3 × 3 mm scanning area, VD measurements in the SCP and DCP slabs were made in the five subfields (central, superior, inferior, nasal, and temporal) using an “Early Treatment Diabetic Retinopathy Study” (ETDRS) grid overlay comprising the two inner rings. The software provides a quantitative characterization of vascular information in terms of vessel density (percentage). VD measurements in the SCP slab were provided automatically, while in the DCP slab we used manual segmentation to obtain accurate VD values (Figure S1). Macular FAZ area, perimeter and circularity index were calculated using the ImageJ software (https://imagej.net/Auto_Threshold*).*

OCT angiograms were analyzed in order to highlight qualitative changes in the retinal microvasculature layer-by-layer. Then, OCTA findings were compared with conventional FA and structural SS-OCT scans for further assessment and validation.

To avoid any potential correlation bias, only one eye per participant was selected for quantitative and qualitative analyses. We have chosen the eye with the higher-quality scan.

### Statistical analysis

Statistical analysis was performed using SPSS 26.0 software version. The comparison of OCTA findings was performed using the Mann-Whitney U test for non-normally distributed continuous variables, and by Khi-squared test or Fisher’s exact test as appropriate for the categorical variables. To identify risk factors of HIV microvasculopathy, we performed univariate then multivariate logistic regression. The receiver operating characteristic (ROC) curves were created using binary logistic regression models, and summary statistics. The area under the curve (AUC), sensitivity, and specificity, were calculated from the ROC curve.

A *P* value < 0.05 was considered statistically significant.

## Results

The study included 102 eyes of 102 HIV-positive patients who met the study criteria and a control group of 100 healthy subjects (100 eyes).

Concerning the HIV-positive group, 72 patients were male and 30 patients were female, with a sex ratio of 2.4 and the mean age was 37.13 ± 7.43 (range 23–53) years.

The mean time from HIV infection diagnosis was 7.24 ± 6.49 (range 0–25) years. Eighty-nine patients (87.25%) were receiving HAART at the time of study recruitment.

Mean HIV RNA plasma level was 50806.4 ± 155850.93 (range 0–981.000) copies/ml and mean CD4 + count was 438.3 ± 381.5 (range 17–2065) cells/mm3, at the time of study enrollment. The overall prevalence of virological failure was 44.1% (45/102) and the CD4 cell count was below 200 cells/mm3 in 17 patients (16.6%).

Based on fundus examination and/or FA, six patients (5.88%) were diagnosed with HIV retinopathy.

Concerning the control group, 65 patients were male and 35 patients were female, with a mean age of 35.83 ± 6.1 years (range 23–49 years).

There were no statistically significant differences between both groups in terms of age, gender, BCVA, IOP, and SE. Demographic and clinical features are summarized in Table 1.

**Table 1:** Demographic and clinical features of HIV-positive patients and healthy controls

	HIV (*n* = 102)	Control (*n* = 100)	*p*-value
Age (years), mean ± SD	37.13 ± 7.43	36.83 ± 6.1	0.167
Gender (M/F)	72/30	65/35	0.23
BCVA (logMAR), mean ± SD	0.02 ± 0.04	0.01 ± 0.03	0.631
IOP (mmHg), mean ± SD	12.33 ± 1.36	12.56 ± 1.02	0.845
Spherical equivalent (dpt)	0.05 ± 0.28	0.04 ± 0.16	0.811
Time from diagnosis (years), mean ± SD	7.24 ± 6.49	-	-
HIV RNA (copies/ml), mean ± SD	50806.4 ± 155850.93	-	-
CD + 4 T cell count (cells/ mm3), mean ± SD	438.3 ± 381.5	-	-

BCVA: Best-corrected visual acuity; IOP: Intraocular pressure; HIV: human immunodeficiency virus, p value was obtained after comparing the two groups using the T-Student test

### Descriptive analysis

#### Qualitative analysis of OCTA images

**Fig. 1:**
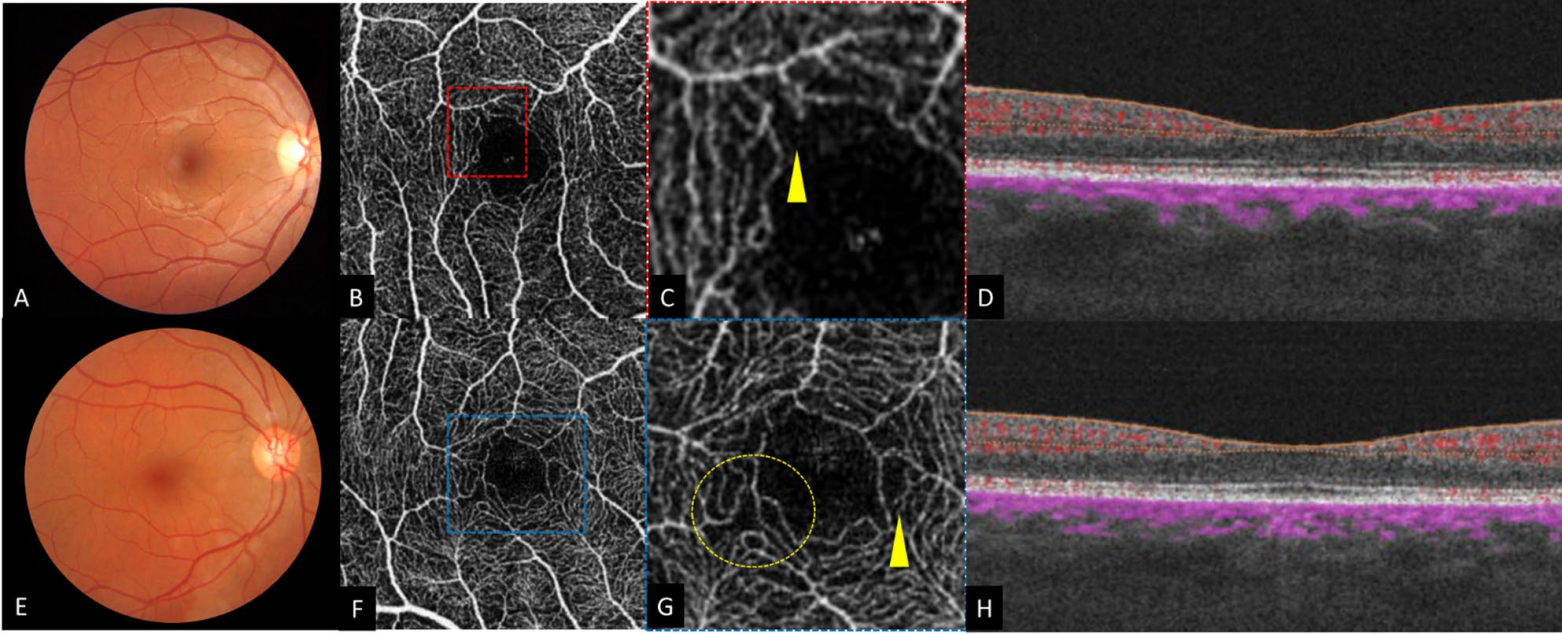
OCTA images featuring HIV-related microangiopathy. A 26-year-old man diagnosed with HIV infection since 3 years (HIV RNA = 79 copies/ml, CD4 nadir counts = 690 cells/ mm3): (**A**) Fundus photograph of the right eye clinically normal (**B**) En face OCTA of the superficial retinal layers demonstrating a disruption of the foveal avascular zone (FAZ) outlines more visible the magnified rectangle (**C**) (yellow arrow), (**D**) Corresponding OCT B-scans show normal retinal layers. A 39-year-old man diagnosed with HIV infection since 18 years (HIV RNA = 51 copies/ml, CD4 nadir counts = 667 cells/ mm3): (**E**) Fundus photograph of the right eye clinically normal, (**F**) En face OCTA of the superficial retinal layers showing capillary loops (yellow circle), intercapillay spacing and vascular telangiectasias (yellow arrow) more visible in the magnified rectangle (**G**), (**H**) Corresponding OCT B-scans show normal retinal layers

**Fig. 2:**
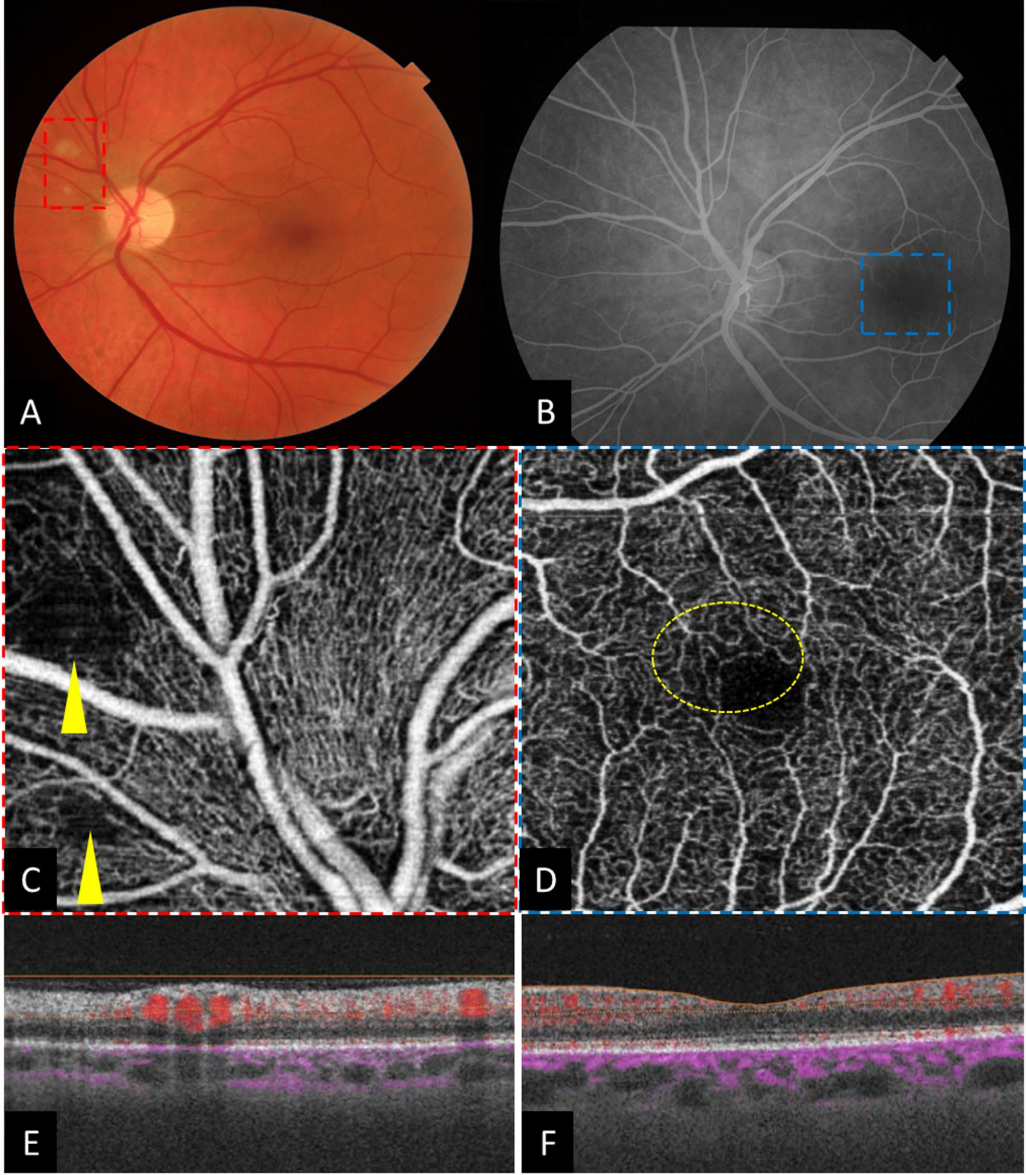
A 46-year-old man diagnosed recently with HIV infection and not receiving yet the highly active antiretroviral therapy (HIV RNA = 198000 copies/ml, CD4 nadir counts = 144 cells/ mm3): (**A**) Color fundus photograph showing the presence of cotton-wool spots superonasal to the optic nerve head, (**B**) Conventional FA with normal appearance, (**C**) En face OCTA image of the corresponding area (red rectangle) showing the presence of hyporeflectivity (yellow arrow), (**D**) En face OCTA of the superficial retinal layers showing capillary loops and intercapillay spacing (yellow circle), (**E**) The corresponding OCT B-scan passing through the cotton-wool spots revealing hyperreflectivity of the retinal nerve fiber layer resulting from the ischemic edema and shadowing effect resulting in the dark area on OCTA, (**F**) Corresponding macular OCT B-scans show normal retinal layers

OCTA images of HIV-positive patients demonstrated perifoveal capillary changes with FAZ outlines disruption in 3 eyes (2.9%), capillary loops in 77 eyes (75.49%), telangiectasia in 65 eyes (63.72%), intercapillary spacing in 88 eyes (86.27%) and capillary tortuosity in 3 eyes (2.9%) (Fig. 1). Those OCTA changes were significantly more prevalent in the SCP. Both observers reached an agreement in identifying these qualitative changes in all eyes. Within cotton-wool spot areas, OCTA demonstrated the presence of hyposignal areas corresponding to capillary flow voids at the SCP (Fig. 2). Only six patients were diagnosed with HIV-related retinopathy. In these patients, OCTA highlighted the presence of microaneurysms in 4 eyes, intraretinal hemorrhages in 2 eyes, and cotton-wool spots in 7 eyes. It is noteworthy to mention that all these patients showed perifoveal capillary changes in OCTA images.

**Fig. 3:**
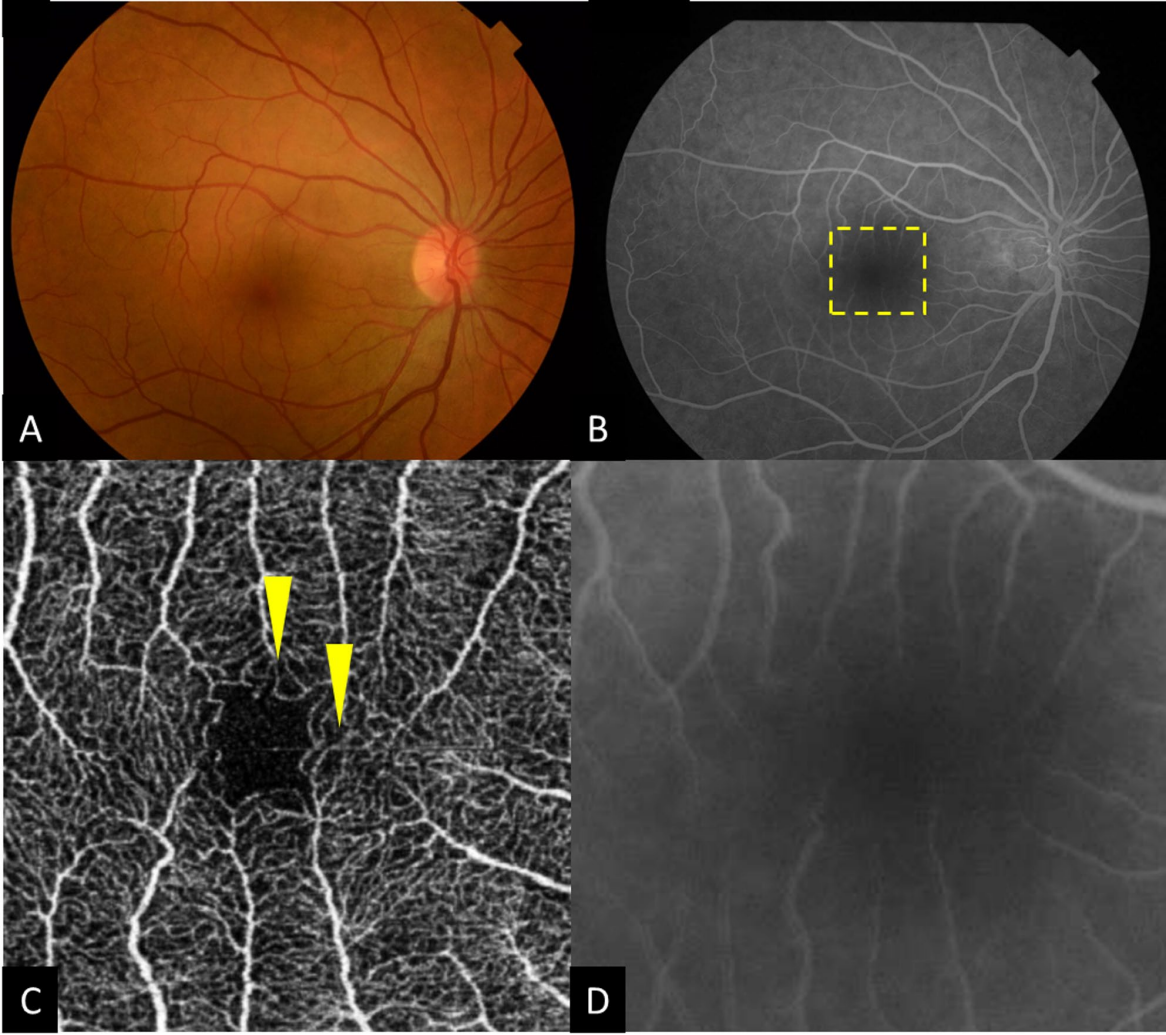
Comparison of OCTA and FA for detection of HIV-related microangiopathy. A 35-year-old man diagnosed with HIV infection since 3 years (HIV RNA < 20 copies/ml, CD4 nadir counts = 383 cells/ mm3): (**A**) Fundus photograph of the left eye clinically normal, (**B**) Conventional FA showing no features of HIV-related microangiopathy, (**C**) En face OCTA of the superficial retinal layers showing increased irregularities of FAZ outlines, intercapillary spacing and capillary loops (yellow arrowheads) suggestive of HIV-related microangiopathy, (**D**) Magnified scans of FA do not reveal any subtle vascular changes

Among the 96 HIV-positive patients without detectable retinal vascular anomaly on funduscopy nor FA (no HIV retinopathy), 72 eyes (75%) showed microvascular changes on en face OCTA (Fig. 3).

#### Quantitative analysis of OCTA images

The mean parafoveal VD was significantly reduced in the SCP (47.52% vs. 48.37%, *p* < 0.001) and DCP (49.38% vs. 50.09%, *p* < 0.001) in HIV-positive group compared with control group. (Table 2).

**Table 2:** Measurement of vessel Densities in the superficial and deep retinal plexus on OCT Angiography in HIV-positive patients and healthy controls

Parameters (%)	HIV (*n* = 102)	Control (*n* = 100)	*p*-value
Mean SCP-VD	47.52 ± 2.13	48.37 ± 1.09	**< 0.001**
Central SCP-VD	20.33 ± 4.41	23.86 ± 2.36	**< 0.001**
Superior SCP-VD	48.92 ± 2.79	49.62 ± 1.83	**0.003**
Inferior SCP-VD	48.63 ± 2.9	49.47 ± 1.71	**< 0.001**
Temporal SCP-VD	46.69 ± 2.56	47.76 ± 2.13	**< 0.001**
Nasal SCP-VD	45.83 ± 2.94	46.61 ± 1.55	**0.001**
Mean PCP-VD	49.38 ± 2.2	50.09 ± 1.49	**0.004**
Central PCP-VD	18.13 ± 4.52	19.38 ± 3.43	**0.002**
Superior PCP-VD	50.98 ± 3.53	51.74 ± 2.46	**0.012**
Inferior PCP-VD	50.78 ± 3.53	51.44 ± 2.5	**0.031**
Temporal PCP-VD	47.34 ± 2.74	48.15 ± 2.05	**0.001**
Nasal PCP-VD	48.42 ± 2.82	49.04 ± 2.53	**0.02**

SCP: Superficial capillary plexus; VD: vessel density; PCP: Deep capillary plexus. p value was obtained after comparing the two groups using the T-Student test

The mean FAZ area in the SCP was significantly larger in HIV-positive group compared with healthy controls (349.42 µm^3^vs. 284.05 µm^3^, *p* < 0.001). The circularity index of FAZ was significantly decreased in the HIV-positive group (0.71 vs. 0.84, *p* < 0.001) (Figure S2).

### OCTA analysis

In HIV-positive group, there was a significant positive correlation between FAZ area and the time from the diagnosis (*r* = 0.141, *p* = 0.042). FAZ circularity index was also significantly correlated with the time from the diagnosis (*r* = -0.25, *p* < 0.001). FAZ parameters showed no significant correlation with HIV RNA viral load (Table S1).

Mean SCP-VD and DCP-VD had a significant negative correlation with the time from the diagnosis (*r*= -0.257, *p* < 0,001 and *r* = -0.17, *p* = 0.017; respectively). We noted also a negative significant correlation with HIV RNA viral load and SCP-VD in all the quadrants (Table S2).

ROC curves were used to assess the ability of each parameter of OCTA in diagnosing eyes with HIV-related non-infectious retinal microvasculopathy. The area under the curve was 0.748 (*p* = 0.003) for the temporal SCP-VD and 0.834 (*p* < 0.001) for the superior DCP-VD.

Therefore, a temporal SCP-VD ≤ 47.73% would predict the presence of HIV-related microvasculopathy with a sensitivity of 100% and a specificity of 58.6%. Similarly, a superior DCP-VD ≤ 50.25% would predict the presence of HIV-related microvasculopathy with a sensitivity of 100% and a specificity of 69.1% (Figure S3).

### Subgroups analysis

The subgroups analysis revealed that patients with virologic failure have significantly more microvascular changes on OCTA (*p* < 0.001), with a decreased mean SCP-VD (*p* = 0.03). All patients with a CD4 count below 200/mm3 have retinal microvascular changes on OCTA (*p* = 0.037) and a larger FAZ (*p* = 0.026) (Table S3).

### Multivariate analysis

A multivariate logistic regression analysis was performed to identify independent factors associated with OCTA vascular abnormalities. The variables entered into the model were age, CD4 cell count, viral load and duration of HIV infection. After adjustment for potential confounders, the duration of HIV infection emerged as the only independent predictor of OCTA vascular changes (adjusted OR = 1.183; 95% CI: 1.016–1.378; *p* = 0.033). The remaining variables did not reach statistical significance. (Table 3).

**Table 3:** Results of the multivariate logistic regression analysis

	Vascular Changes in OCTA			
	*p*-value	Adjusted Odds Ratio	Confidence interval	
			**Lower bound**	**Upper bound**
Age (years)	0.114	1.073	0.983	1.17
CD + 4 T cell count (cells/ mm3)	0.542	1	0.999	1.002
HIV RNA (copies/ml)	0.988	1	0.94	1.065
Virologic failure	0.996	0	0	
Time from diagnosis (years)	**0.033**	**1.183**	**1.016**	**1.378**

HIV: human immunodeficiency virus; OCTA: OCT Angiography

## Discussion

The pathogenesis of HIV-related noninfectious retinal vasculopathy remains poorly understood [5]. Several prior clinical studies have found retinal changes in HIV positive patients, including cotton wool spots, intraretinal hemorrhages, and microaneurysms, which are mostly ascribed to focal ischemia affecting small vessels and hemodynamic abnormalities [8, 9]. The ultrastructural study analysis of autopsy cases with HIV disease have confirmed those retinal microvascular changes [9]. Thus, investigators had tried to explore retinal vascular calibers measurements through fundus photographs which have shown a reduced arteriolar-to-venular ratio among individuals with HIV infection [10, 11]. However, studies focusing on retinochoroidal microvascular alterations in HIV infection using OCTA were limited [8, 12–14]. The purpose of this index study was to describe qualitative alterations of HIV-related retinopathy using OCTA and to evaluate the vascular density changes in HIV-positive patients group, when compared with healthy controls. To best of our knowledge, this is the largest study to describe qualitative and quantitative retinal microvascular alterations in HIV-related microvasculopathy. The first study OCTA was conducted by Agarwal et al. [8] who have described different morphological features of HIV-related retinopathy including vascular loops, increased intercapillary spacing and telangiectasias in the SCP. Such findings were also identified in patients without clinical HIV microangiopathy which emphasize the enhanced sensitivity of OCTA in detecting HIV-related microvasculopathy. Our results were consistent with this study. In fact, we found that even patients without any abnormal retinal findings on FA, showed features of HIV-related microangiopathy on en face OCTA. These features included capillary loops, telangiectasias, intercapillary spacing, capillary tortuosity and also disruption of the FAZ outlines which has not been reported in previous studies. Most of the retinal microvascular changes were observed in the SCP. Compared to Argawel et al. [8], we observed a higher proportion of patients showing HIV-related microangiopathy features on OCTA without any clinical detectable retinal abnormalities. This could be attributed to the significant variability in the immunovirologic status of patients and variations in the duration of disease progression. Statistical analysis comparing patients with HIV retinopathy and those without HIV retinopathy on FA was not possible due to the limited number of HIV retinopathy cases (only 6 patients showed HIV retinopathy features on FA). Our OCTA results align closely with the microvascular alterations reported in ultrastructural studies conducted three decades ago. Anatomopathologists had described retinal vascular abnormalities similar to those observed in diabetic retinopathy, such as pericyte loss, endothelial cell edema, thickening of the capillary basement membrane, and ischemic maculopathy [9, 15].

Quantitative analysis of OCTA images revealed that vessel densities were significantly reduced in all macular sectors in the superficial and deep retinal plexus in HIV group compared to controls. These results agreed well with Esen et al. [12], who reported that HIV could also affect the deeper retinal layer. However, such findings did not concur with the observations made by Agarwel et al. and Akmaz et al. who noted decreased vessel densities only in the in the SCP in HIV-positive patients when compared to controls [8, 16].

The analysis of the FAZ revealed that FAZ are larger and less circular among HIV-positive patients when compared with healthy controls. This align closely with the result of Agarwal et al. [8] but did not with Esen et al. [12]. This could be explained by the variability of FAZ area among healthy individuals [17].

The current study showed that virologic failure and higher plasma HIV RNA levels were associated with decreased vessel densities in the SCP. Lower CD4 counts was associated with larger FAZ. It is noteworthy to mention that both HIV virologic failure and lower CD4 counts were significantly associated with features of HIV-related microangiopathy on en face OCTA. These results agree well with the literature [8, 12]. However, unlike previous reports, our multivariate analysis revealed that only HIV diagnosis duration was significantly associated with features of HIV-related microangiopathy on en face OCTA. This supports the suggestion that, even with successful HIV suppression and immunity restoration, HIV- positive patients may have a persistent of inflammatory state leading to an accelerated neuroretinal degeneration which could be the potential risk for retinal microvascular alterations [13, 18, 19].

With regard to the diagnostic capability of OCTA parameters for distinguishing eyes with HIV microangiopathy from eyes without HIV microangiopathy, the results showed that the temporal SCP-VD (AUC = 0.816) and superior DCP-VD (AUC = 0.834) have the best diagnostic ability. In fact, a temporal SCP-VD ≤ 47.73% predicts the presence of HIV-related microvasculopathy with a sensitivity of 100% and a specificity of 58.6%. Similarly, a superior DCP-VD ≤ 50.25% predicts the presence of HIV-related microvaculopathy with a sensitivity of 100% and a specificity of 69.1%. This is the first study to provide thresholds in diagnosing HIV-related microangiopathy.

Several strengths of this study should be highlighted. First, it included a relatively large cohort of HIV-positive patients and a control group of healthy subjects matched for age and sex, allowing a reliable comparison of retinal microvascular parameters between groups. Second, the use of OCTA, a non-invasive imaging technique, enabled detailed quantitative assessment of macular microvascular alterations. Nevertheless, some limitations should be acknowledged. The cross-sectional design of the study does not allow evaluation of the temporal progression of retinal microvascular changes or causal relationships. Furthermore, OCTA measurements may be influenced by image quality and segmentation errors, although careful acquisition and analysis were performed to minimize these potential biases.

Further longitudinal studies with larger sample size are needed to clarify the long-term effect of HIV on retinal microvasculature and the associations with patients’ immunovirologic status and the disease duration.

### Conclusion

The findings of our study indicate that OCTA may be a useful tool in detecting the presence of HIV-related microangiopathy which had been identified as an independent prognostic factor for mortality. Subsequently, it becomes essential for ophthalmologist to screen HIV-related microvasculopathy features among HIV-positive patients.

## Supplementary Information

Below is the link to the electronic supplementary material.

Supplementary Material 1

## Data Availability

The data that support the findings of this study are available from the corresponding author upon reasonable request.

## Abbreviations

ART: Antiretroviral therapy
BCVA: Best-corrected visual acuity
DCP-VD: Deep capillary plexuses vessel density
ETDRS: Deep capillary plexuses vessel density
FA: Fluorescein angiography
FAZ: Foveal avascular zone
HAART: Highly active antiretroviral therapy
HIV: Human Immunodeficiency Virus
ILM: Internal limiting membrane
INL: Inner nuclear layer
IOP: Intraocular pressure
IPL: Inner plexiform layer
OCT: Image quality index
OCT: Optical coherence tomography
OCTA: Optical coherence tomography angiography
SCP-VD: Superficial capillary plexuses vessel density
SE: Spherical equivalent
SS-OCT: Swept source Optical coherence tomography
VD: Vessel density
