# Supplementary material for: Analysis of macular microvasculature changes in human immunodeficiency virus infection using swept-source OCT angiography
Source: BMC Ophthalmol. 2026 Apr 18;26:197. doi: 10.1186/s12886-026-04822-9 (PMC13094047; doi:10.1186/s12886-026-04822-9)
Supplement: Supplementary file 1 — Supplementary Material 1 [file 12886_2026_4822_MOESM1_ESM.docx]

**Supplemental material.**

**Table S1. Correlations between FAZ parameters and demographic and clinical features of HIV-positive patients**

|  | | FAZ parameters | |  |
| --- | --- | --- | --- | --- |
|  | | **Area** | **perimeter** | **Circularity index** |
| Age (years) | | r = 0.299, **p<0.001** | r = 0.145, **p=0.042** | r = 0.15, **p=0.03** |
| Gender | **Male, mean± SD** | 333.88 ±106.53 | 2.40 ±0.40 | 0.700 ±0.127 |
|  | **Female, mean± SD** | 386.71 ±116.25 | 2.51 ±0.44 | 0.756 ±0.121 |
| *p*-value | | **0.006** | 0.135 | **0.002** |
| HIV RNA (copies/ml) | | r = -0.11,  p = 0.121 | r = -0.085,  p = 0.236 | r = -0.063, p=0.37 |
| CD+4 T cell count (cells/ mm3) | | r = -0.069,  p = 0.336 | r = -0.097, p = 0.175 | r = 0.07, p=0.278 |
| Time from diagnosis (years) | | r = 0.141, **p=0.042** | r = -0.002, p = 0.977 | r = -0.25, **p<0.001** |

*FAZ: foveal avascular zone; HIV: human immunodeficiency virus. The r and p values were obtained by a Pearson correlation test.*

**Table S2. Correlations between OCTA measurements and demographic and clinical features of HIV-positive patients**

*SCP: Superficial capillary plexus; VD: vessel density; PCP: Deep capillary plexus; HIV: human immunodeficiency virus. The r and p values were obtained by a Pearson correlation test.*

|  | | OCT Angiography Vascular densities | | | | | | | | | | | | |
| --- | --- | --- | --- | --- | --- | --- | --- | --- | --- | --- | --- | --- | --- | --- |
|  | | **SCP-VD** | | | | | | **DCP-VD** | | | | | | |
|  | | **Mean** | **Temporal** | **Nasal** | **Inferior** | **Superior** | **Central** | **Mean** | **Temporal** | **Nasal** | **Inferior** | | **Superior** | **Central** |
| Age (years) | | r = -0.052, p=0.47 | r = 0.017, p=0.8 | r = -0.001, p=0.993 | r = 0.006, p=0.928 | r = -0.023, p=0.746 | r = -0.349, **p<0.001** | r =-0.052, p=0.471 | r = -0.06, p=0.35 | r = 0.047, p=0.507 | r = 0.031, p=0.66 | r = -0.06, p=0.37 | | r = -0.257, **p<0.001** |
| Gender | **Male** | 9.61 ±2.11 | 46.6 ±2.40 | 45.81 ±2.49 | 48.69 ±2.6 | 48.82 ±2.67 | 20.86 ±4.37 | 13.71 ±2.19 | 47.09 ±2.73 | 48.4 ±2.96 | 50.57 ±3.44 | 50.83 ±3.67 | | 18.53 ±4.54 |
|  | **Female** | 10.32 ±2.06 | 46.91 ±2.92 | 45.88 ±3.85 | 48.49 ±3.53 | 49.16 ±3.08 | 19.08 ±4.3 | 13.68 ±1.9 | 47.94 ±2.7 | 48.4±2.4 | 51.28 ±3.7 | 51.35 ±3.16 | | 17.16 ±4.33 |
| *p*-value | | **0.026** | 0.412 | 0.114 | 0.722 | 0.853 | **0.018** | 0.815 | 0.102 | 0.948 | 0.214 | 0.330 | | 0.066 |
| HIV RNA (copies/ml) | | r = -0.027, **p=0.009** | r = -0.299, **p<0.001** | r = -0.489, **p<0.001** | r = -0.363, **p<0.001** | r = -0,314, **p<0,001** | r = 0.039, p=0.586 | r = 0.033, p=0.649 | r = -0.062, p=0.384 | r =-0.186, **p=0.009** | r = 0.045, p=0.528 | r = -0.061, p=0.394 | | r = -0.045, p=0.525 |
| CD+4 T cell count (cells/ mm3) | | r = -0.056, p=0.43 | r = 0.156, p=0.028 | r = 0.239, **p=0.001** | r = 0.217, **p=0.002** | r = 0,343, **p<0,001** | r = 0.118, p=0.098 | r = 0.045, p=0.524 | r = 0.106, p=0.138 | r = 0.06, p=0.385 | r = 0.066, p=0.359 | r = 0.108, p=0.131 | | r = 0.178, **p=0.012** |
| Time from diagnosis (years) | | r = -0.257, **p<0.001** | r = -0.151, **p=0.034** | r = -0.127, p=0.075 | r = -0.097, p=0.173 | r = -0,148, **p=0,037** | r = -0.219, **p=0.002** | r = -0.17, **p=0.017** | r = -0.06, p=0.37 | r = 0.102, p=0.154 | r = -0.02, p=0.738 | r = 0.054, p=0.449 | | r = -0.25, **p<0.001** |

**Table S3. Subanalysis group**

|  | | HIV virologic failure | | | CD+4 T cell count (cells/ mm3) | | |
| --- | --- | --- | --- | --- | --- | --- | --- |
|  | | **Yes** | **No** | **p** | **<200** | **>200** | ***p*-value** |
| FAZ | **Area** | 353.32 ±110.71 | 345.42 ±109.95 | 0.574 | 311.23 ±104.42 | 357.18 ±109.89 | **0.026** |
|  | **Perimeter** | 2.44 ±0.43 | 2.44 ±0.4 | 0.877 | 2.31 ±0.37 | 2.46 ±0.41 | 0.43 |
|  | **Circularity index** | 0.725 ±0.115 | 0.706 ±0.137 | 0.301 | 0.697 ±0.161 | 0.717 ±0.12 | 0.415 |
| Mean SCP-DV | | 46.11  ±2.21 | 47.75±1.94 | **0.03** | 46.14 ±2.51 | 46.61 ±2.03 | 0.198 |
| Mean DCP-DV | | 48.92±1,83 | 49.23 ±2.33 | 0.709 | 49.04 ±1.68 | 49.43 ±2.21 | 0.711 |
| Vascular changes in OCTA | | 45 (100) | 41 (71.92) | **<0.001** | 17 (100) | 69 (81.17) | **0.037** |

*SCP: Superficial capillary plexus; VD: vessel density; PCP: Deep capillary plexus; HIV: human immunodeficiency virus; FAZ: foveal avascular zone; OCTA: OCT Angiography. P values were obtained after comparing the two groups using th T-Student test*


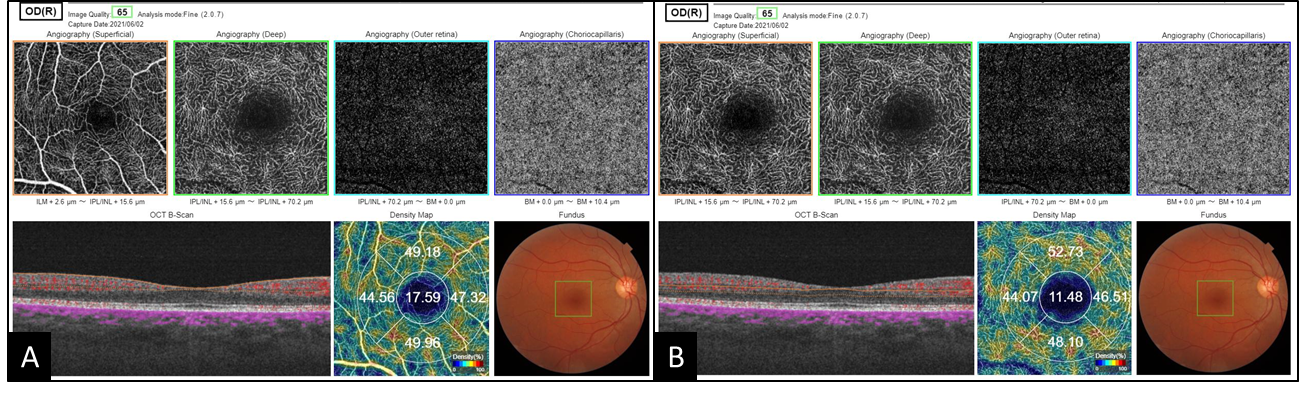


**Figure S1:** OCTA images (3 × 3mm) of an HIV-positive patient showing the measurements of vessel densities of (A) superficial capillary plexus (SCP) and (B) deep capillary plexus (DCP) in the foveal (central) and parafoveal subfields (superior, temporal, inferior, and nasal)


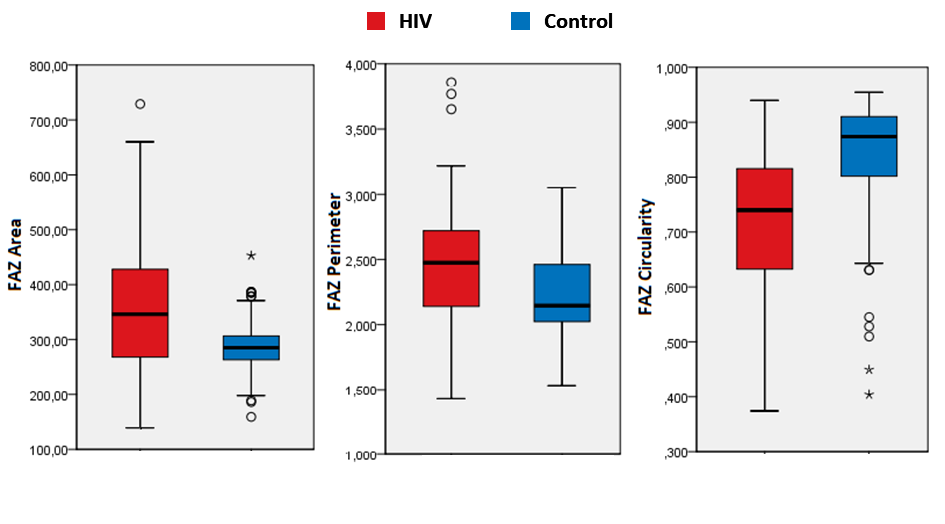


**Figure S2:** Box plot showing a significant differences in the foveal avascular zone (FAZ) area, perimeter and circularity between HIV-positive group and controls.


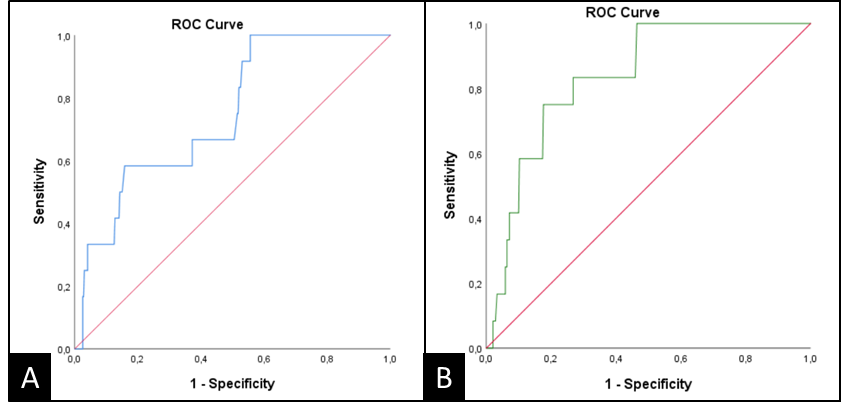


**Figure S3:** Area under the curve (AUC) for distinguishing between eyes with HIV microangiopathy from eyes without HIV microangiopathy, (A) AUC for temporal SCP-VD and (B) AUC for superior DCP-VD.
